# Is homologous recombination really an error-free process?

**DOI:** 10.3389/fgene.2014.00175

**Published:** 2014-06-11

**Authors:** Josée Guirouilh-Barbat, Sarah Lambert, Pascale Bertrand, Bernard S. Lopez

**Affiliations:** ^1^CNRS, UMR 8200, Institut de Cancérologie Gustave Roussy, Équipe Labélisée, Université Paris-Sud, «LIGUE 2014»Villejuif, France; ^2^Institut Curie, CNRS, UMR 3348Orsay, France; ^3^CEA DSV, UMR 967 CEA-INSERM-Université Paris Diderot-Université Paris Sud, Institut de Radiobiologie Cellulaire et MoléculaireFontenay-aux-Roses, France

**Keywords:** Homologous recombination, mutagenesis, DNA double strand break repair, replication stress, genetic variability, genetic instability

## Abstract

Homologous recombination (HR) is an evolutionarily conserved process that plays a pivotal role in the equilibrium between genetic stability and diversity. HR is commonly considered to be error-free, but several studies have shown that HR can be error-prone. Here, we discuss the actual accuracy of HR. First, we present the product of genetic exchanges (gene conversion, GC, and crossing over, CO) and the mechanisms of HR during double strand break repair and replication restart. We discuss the intrinsic capacities of HR to generate genome rearrangements by GC or CO, either during DSB repair or replication restart. During this process, abortive HR intermediates generate genetic instability and cell toxicity. In addition to genome rearrangements, HR also primes error-prone DNA synthesis and favors mutagenesis on single stranded DNA, a key DNA intermediate during the HR process. The fact that cells have developed several mechanisms protecting against HR excess emphasize its potential risks. Consistent with this duality, several pro-oncogenic situations have been consistently associated with either decreased or increased HR levels. Nevertheless, this versatility also has advantages that we outline here. We conclude that HR is a double-edged sword, which on one hand controls the equilibrium between genome stability and diversity but, on the other hand, can jeopardize the maintenance of genomic integrity. Therefore, whether non-homologous end joining (which, in contrast with HR, is not intrinsically mutagenic) or HR is the more mutagenic process is a question that should be re-evaluated. Both processes can be “Dr. Jekyll” in maintaining genome stability/variability and “Mr. Hyde” in jeopardizing genome integrity.

## Introduction

Genomes are routinely challenged with exogenous or endogenous insults of enzymatic, chemical or physical origins. These DNA alterations can generate genetic instability, leading to cell death, senescence, developmental abnormalities and tumor initiation and progression. However, while it is vital to maintain genomic stability, genetic diversity is essential to physiological processes, such as the generation of the immune repertoire or the mixing of parental alleles during meiosis. Additionally, the absence of genetic diversity would constitute an evolutionary dead end. Thus, DNA repair should maintain genomic stability and allow for genetic diversity. Therefore, the accuracy of DNA repair processes is an essential issue.

Homologous recombination (HR) is a process that is conserved in all organisms, playing an essential and pivotal role in genome stability and plasticity. Notably, HR is involved in the reactivation of replication forks that have been blocked and in the repair of DNA double strand breaks (DSBs) (reviewed in Haber, 2014).

Replication fork progression is routinely challenged by diverse exogenous or endogenous stresses, which ultimately leads to replication fork stalling, collapse or breakage, and triggers the DNA damage response (DDR) (Hyrien, 2000; Lambert and Carr, 2005, 2013; Tourriere and Pasero, 2007; Aguilera and Garcia-Muse, 2013). Failures in chromosome replication are thus a primary source of genetic instability. Consistently, in many organisms, including yeast and human cells, both slowing down and blocking fork progression are associated with chromosome breakage and genome rearrangement (reviewed in Aguilera and Gomez-Gonzalez, 2008; Branzei and Foiani, 2010). Moreover, impediments to fork progression might also challenge the completion of DNA replication, resulting in mitotic defects and multipolar mitotic cells, which then lead to uneven chromosome segregation and thus amplifying the genome instability to the whole genome, including fully replicated regions (Wilhelm et al., 2014). Consistently with the existence of endogenous replication stresses, DDR activation resulting from spontaneous endogenous replication stress has also been detected in the early stages of carcinogenesis and senescence (Bartkova et al., 2005, 2006; Gorgoulis et al., 2005; Halazonetis et al., 2008; Gorgoulis and Halazonetis, 2010).

DSBs are harmful lesions that are produced through exposure to exogenous treatments, such as ionizing radiation (IR), byproducts of endogenous cellular metabolisms and, importantly, replication forks arrest (Seigneur et al., 1998; Featherstone and Jackson, 1999; Saintigny et al., 2001; Rothkamm and Lobrich, 2003; Mahaney et al., 2009). DSBs can trigger profound genomic rearrangements or, in contrast, generate genetic diversity in essential biological processes. In the latter case, programmed DSBs are physiologically produced through controlled cellular enzymes during meiotic differentiation, mating-type switching in *Saccharomyces cerevisiae* or in V(D)J and class switch recombination, which ensures the diversity of the immune response (reviewed in Haber, 1992; Jung and Alt, 2004; Lieber et al., 2004; Rooney et al., 2004; Dudley et al., 2005; Buard and de Massy, 2007).

**Figure 1:**
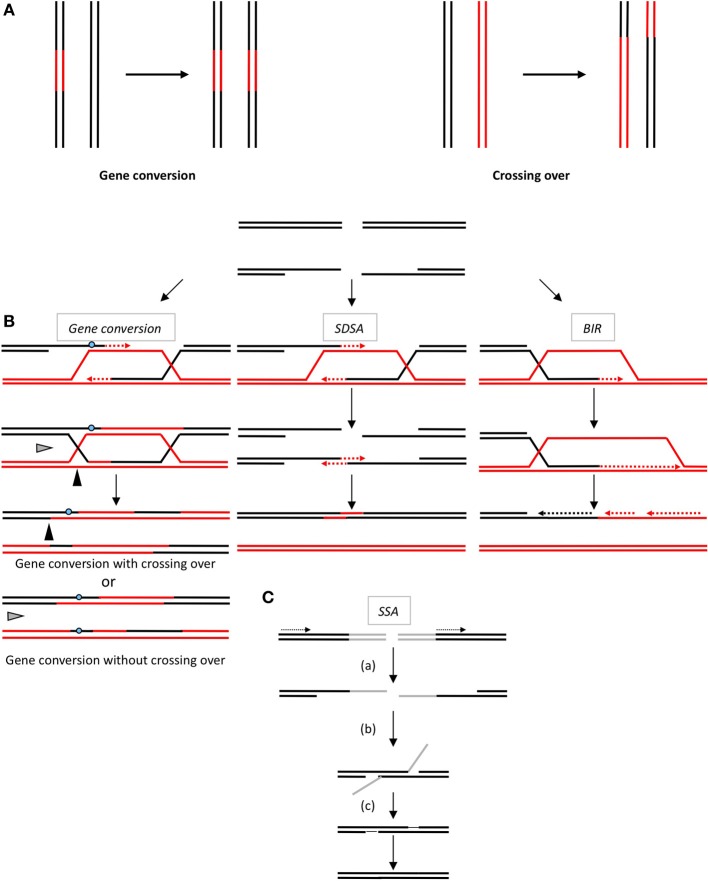
**(A)** The products of HR. Gene conversion (left panel) leading to non-reciprocal exchange of a DNA sequence (in red). Crossing over (right panel): reciprocal exchanges of adjacent sequences (black and red). Note that gene conversion can be associated with or without crossing over. **(B)** The double-strand break repair models through HR. Left panel: Gene conversion. After resection, the single-stranded 3′-tail invades a homologous, intact double-stranded DNA, forming a D-loop (displacement loop). This process tolerates limited imperfect sequence homologies, thus creating heteroduplex intermediates bearing mismatches (blue circle). The invading 3′-end primes DNA synthesis, which then fills in the gaps. The cruciform junctions (Holliday junctions, HJ) migrate. Resolution (or dissolution) of the HJ occurs in two different orientations (black or gray triangles), resulting in gene conversion either with or without crossing over. Middle panel: Synthesis-dependent strand annealing. Initiation is similar to that of the previous model, but the invading strand de-hybridizes and re-anneals at the other end of the injured molecule; no HJ is formed. Right panel: Break-induced replication (BIR). The initiation is similar to that of the previous models, but the synthesis continues over longer distances on the chromosome arms, even reaching the end of the chromosome. Here, there is neither resolution of the HR nor crossover. **(C)** Single-strand annealing (SSA). When a double-strand break is generated between two homologous sequences in tandem in the same orientation (dotted arrows), an extended single-strand resection (a) reveals two complementary DNA strands that can hybridize (b). (c) Resolution of the intermediate and gap filling complete the repair, leading to the deletion of the intergenic sequences between the initial repetitions.

Two primary strategies are used to repair DSBs: (1) HR, which requires a sequence-homologous partner and, in fact, corresponds to different processes involving both common and distinct mechanisms (see below and Figure 1); and (2) NHEJ (non-homologous end joining), which ligates the DNA ends of a DSB without requiring extended homologies (Haber, 2014). Note that a highly mutagenic alternative end-joining pathway (A-EJ) has recently been identified (for review Grabarz et al., 2012; Rass et al., 2012; Betermier et al., 2014).

In most of the literature, HR is described as an error-free process, while NHEJ is described as an error-prone DSB repair process. This statement is largely based on the fact that the mechanism of HR requires the search for a homologous partner to repair DNA, in contrast to NHEJ. Careful examination of the data from the literature might challenge these assumptions, which requires revisiting the current view. Indeed, recent data points to the intrinsic precision of canonical NHEJ (C-NHEJ; KU-Ligase 4-dependent) in contrast to A-EJ. In fact, C-NHEJ is conservative but adaptable, and the accuracy of the repair is dictated by the structure of the DNA ends rather than by the C-NHEJ machinery itself (Grabarz et al., 2012; Rass et al., 2012; Betermier et al., 2014).

Here, in a reciprocal view, we discuss the accuracy of HR and we present several situations of mutagenesis generated by HR. We conclude that HR is a double-edged sword, which on the one hand controls the equilibrium of genomic stability vs. diversity, but on the other hand can jeopardize the maintenance of genomic integrity. The importance of the versatility of HR and its impact on genomic integrity are discussed.

## The products of HR (gene conversion and crossing over) and models

Consistently with the implication of HR in genome stability maintenance, mutant cells that are defective in HR show elevated mutagenesis and genetic instability. However, in contrast, HR can appear as a mutagenic process *per se*, in many situations. Such concepts can be understood when considering the products and molecular mechanisms of HR.

The products of HR are gene conversion (GC: non-reciprocal exchange of genetic material) associated or not with crossing-over (CO: reciprocal exchange of the adjacent sequences) (Figure 1A). Such products can account for genetic diversity or instability arising through HR.

### Models of HR for DSB repair

All HR processes are initiated through the 5′ to 3′-single-stranded resection of double stranded DNA ends, creating a 3′-single-stranded DNA (ssDNA), on which the pivotal RecA/Rad51 recombinase is loaded (Figure 1B). The RecA/Rad51 nucleofilament carries out the subsequent invasion of a homologous DNA duplex that primes DNA synthesis and copies the intact DNA molecule. At this point, the HR processes differ in the processing of the intermediates, leading to either gene conversion, associated or not with crossing-over, or to SDSA (synthesis-dependent strand annealing) and BIR (break-induced replication) (Figure 1B). In addition, an alternative process (SSA, single-strand annealing) is also initiated by resection; however, the following step does not require Rad51 nor strand invasion of an intact duplex DNA, but the annealing of two complementary ssDNAs (Figure 1C). SSA is a non-conservative process that systematically leads to the deletion of the intervening sequence between the two interacting DNA molecules (reviewed in Haber, 2014).

### HR and replication forks reactivation

**Figure 2:**
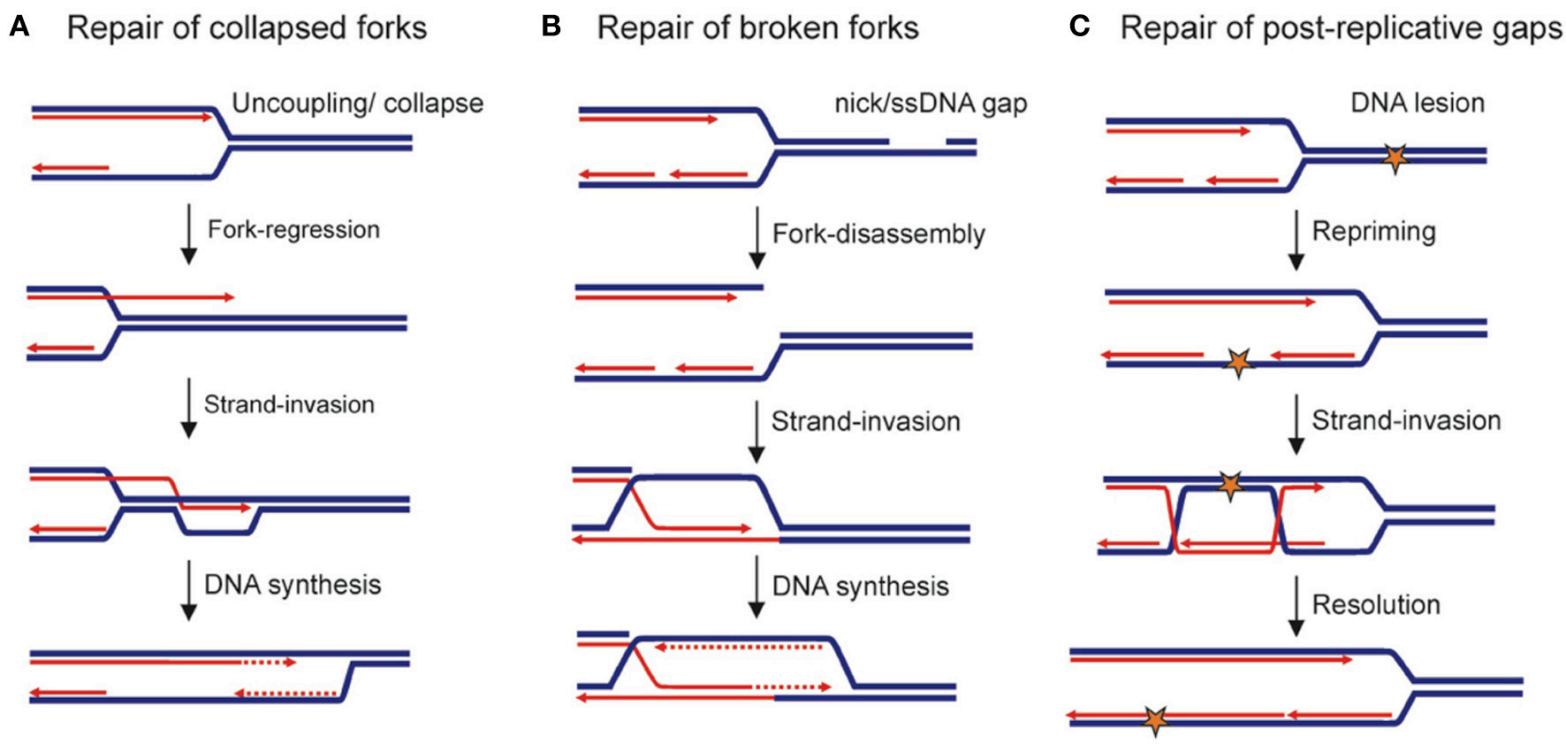
**Replication-maintenance by homologous recombination.** Blue and red lines indicate parental and neo-synthesized strands, respectively. **(A)** Replication-restart following collapse of the replication fork. **(B)** Repair of a broken replication fork. **(C)** Repair of ssDNA gaps that are left behind the moving fork after it has encountered a DNA lesion. Star: DNA damage.

HR contributes to the robustness of DNA replication by multiple mechanisms (Figure 2) and might be viewed as a pathway escorting fork progression (reviewed in Costes and Lambert, 2012) (Figure 2). HR can act either at replication forks or at replicated chromatids to ensure the completion of chromosome duplication. First, HR efficiently seals ssDNA gaps that have been left within replicated chromatids after fork passage through DNA lesions. Second, HR is involved in the recovery of arrested replication forks and has the potential to reassemble a functional replisome. While the mechanism of origin-independent loading of a replisome by HR has been extensively characterized in bacteria, its counterpart in eukaryotic cells has only recently begun to emerge.

Fork passage over a ssDNA nick or gaps in the parental DNA leads to a broken fork, with one of the sister chromatids being disconnected from the fork. Some components of the replisome are thus lost (Roseaulin et al., 2008; Hashimoto et al., 2010; Moriel-Carretero and Aguilera, 2010). HR ensures the repair of such broken forks through a mechanism that is thought to be similar to BIR (Bosco and Haber, 1998; Kraus et al., 2001; Hashimoto et al., 2010). In *Xenopus*, HR-mediated fork repair leads to the reassembly of a replisome (Hashimoto et al., 2012). But BIR that requires most of the components of canonic replisomes (Lydeard et al., 2007, 2010) is highly mutagenic in yeast (Deem et al., 2011). An inter-strand cross-link (ICLs) is a type of lesion that interferes with the progression of replication forks by preventing the unwinding of the parental DNA. ICLs are cleaved by specific nucleases, thus resulting in a broken fork that is then repaired by HR (Long et al., 2011).

Many chromosomal elements can behave as fork obstacles, and it remains unclear whether fork breakages occur systematically. For example, DNA-bound proteins represent more than 1400 potential sites of fork arrest in budding yeast, and HR efficiently rescues replication forks blocked by protein complexes tightly bound to DNA in fission yeast (Ivessa et al., 2003; Lambert et al., 2010; Iraqui et al., 2012). In this case, replication restart is initiated by the loading of HR factors at ssDNA exposed at blocked forks (Mizuno et al., 2009; Lambert et al., 2010). The mechanisms by which HR ensures replication restart remain to be determined. Nevertheless, the resumption of DNA synthesis at inactivated forks via the HR pathway is also mutagenic (see below).

Finally, in addition to rescuing DNA synthesis at replication forks, HR is also involved in the stability and protection of forks that are impeded in their progression. HR defects lead to the accumulation of ssDNA gaps at replication forks, perhaps due to an uncoupling between lagging and leading strand synthesis (Hashimoto et al., 2010). Additionally, resection of neo-synthesized strands has been observed in mammalian and bacterial HR-deficient cells (Courcelle and Hanawalt, 2003; Schlacher et al., 2011). While this fork-stabilizer function of HR during DNA replication appears to be evolutionarily conserved, its importance in ensuring the robustness of DNA replication remains to be established in eukaryotes.

Therefore, because HR acts through multiple pathways at the replication fork or in its vicinity, it should play an essential role in protecting cells against spontaneous replication stress and thus against the resulting genetic instability, as discussed below.

## Role of HR in the maintenance of genome stability

### HR defects result in higher levels of mutagenesis and genetic instability

In all organisms, HR-deficient cells exhibit a higher level of mutagenesis and genome rearrangements, both spontaneous and upon exposure to exogenous genotoxic agents (Quah et al., 1980; Liu et al., 1998; Takata et al., 2001; Thompson and Schild, 2001; Lambert and Lopez, 2002; Popova et al., 2012). These data suggest that HR (like NHEJ) maintains genome stability.

### HR protects mitosis from replication stress

Replication stress covers many events that impact the accuracy of DNA replication and then jeopardize chromosome segregation during mitosis. Low levels of replication stress can generate mitotic defects, including anaphase bridges, supernumerary centrosomes and multipolar mitosis, which then lead to uneven chromosome segregation (Wilhelm et al., 2014). Because HR plays a pivotal role in the resumption of arrested replication forks, defects in HR should thus reveal endogenous replication stress. Consistently, HR-deficient cells are associated with spontaneous slowed replication fork progression (Daboussi et al., 2008; Wilhelm et al., 2014), anaphase bridges (Lahkim Bennani-Belhaj et al., 2010; Laulier et al., 2011b; Rodrigue et al., 2013; Wilhelm et al., 2014), common fragile sites (Ingvarsson et al., 1999; Turner et al., 2002), supernumerary centrosomes (Griffin et al., 2000; Deng, 2002; Kraakman-van der Zwet et al., 2002; Bertrand et al., 2003; Dodson et al., 2004; Daboussi et al., 2005; Katsura et al., 2009; Plo and Lopez, 2009; Rodrigue et al., 2013; Wilhelm et al., 2014), and multipolar mitosis (Wilhelm et al., 2014). Similarly, fission yeast recombination factors are necessary to ensure successful chromosome segregation following the slowdown of fork progression (Bailis et al., 2008).

These data underline the essential role played by HR in protecting genome stability at the interface between replication and mitosis, as reviewed elsewhere (Wilhelm et al., 2014).

## HR: A factor of genetic instability

Because of its intrinsic properties (genetic exchanges through GC and CO), HR can generate genetic instability. More surprisingly, several reports have noted a type of genome instability mediated by micro-homology in an HR-dependent manner. These types of genetic instability were initially assigned to the error-proneness of end joining. Consequently, the actual view on the accuracy of HR has been challenged in many reports.

### HR possesses the intrinsic capacity of genetic modification

**Figure 3:**
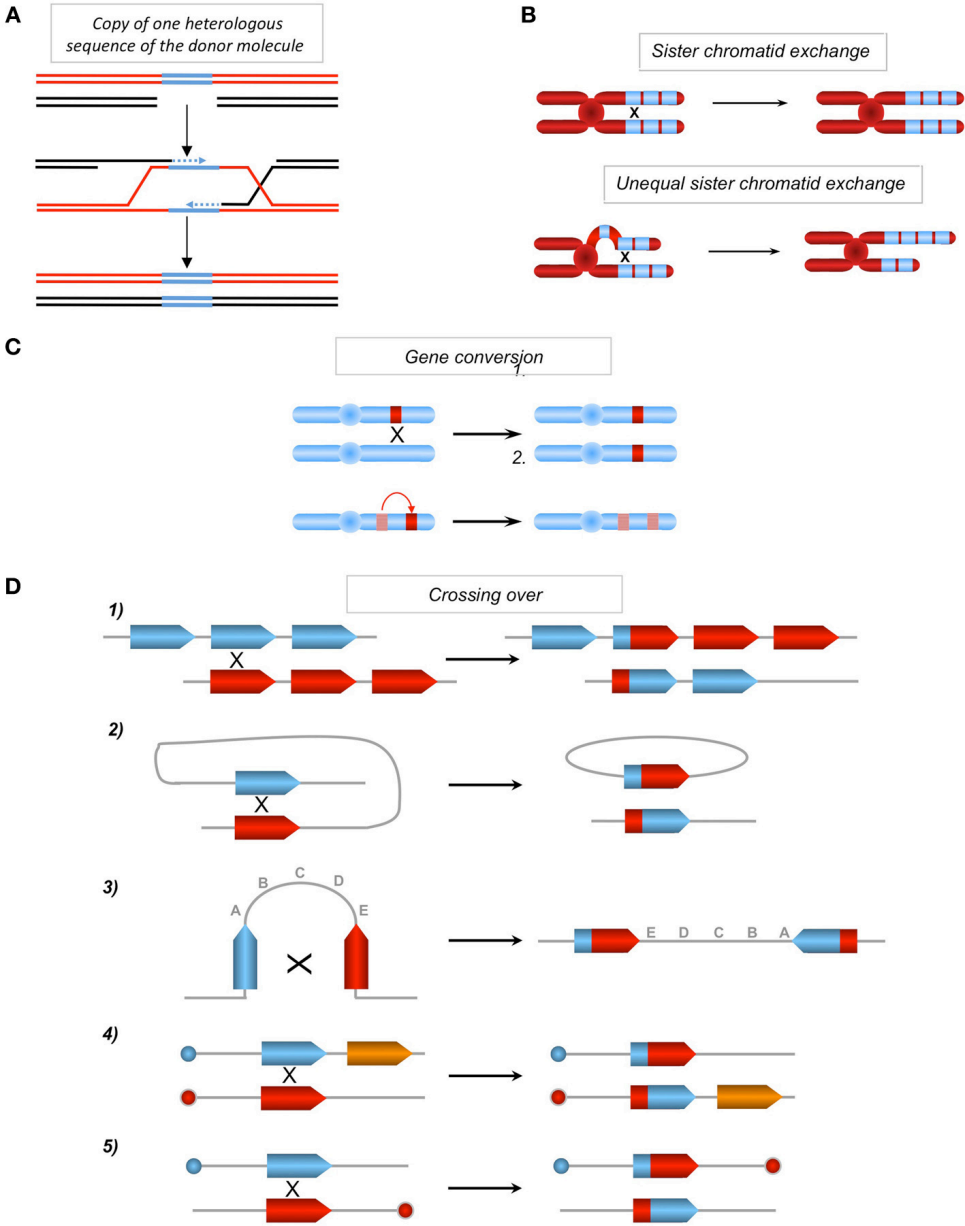
**(A)** Copy of one sequence of the donor absent on the recipient molecule. One of two homologous molecules (red and black) can contain one heterologous sequence (blue). Upon gene conversion or SDSA (see Figure 1) the heterologous (blue) sequence can be copied and transferred from the donor sequence (red) to the homologous recipient sequence (black), resulting in a genetic modification of the recipient sequence. **(B)** Sister chromatid exchanges. Between repeat sequences (blue boxes) without misalignment (upper panel) or with misalignment resulting in unequal sister chromatid exchanges (lower panel) and amplification and loss of genetic material. **(C)** Impact of gene conversion. Non-reciprocal exchange of genetic information between two heteroalleles, leading to a loss of heterozygosity (upper panel) and between a pseudogene (hatched), which often contains nonsense mutations and a gene (in red), leading to the inactivation of the latter (lower panel). **(D)** Chromosomal rearrangements resulting from crossing-over (CO) between repeat sequences. (1) Between homologous sequences on two chromosomes or following unequal sister chromatid exchange on the same chromosome, resulting in the amplification of one molecule and the deletion of the other. (2) Intramolecular CO between two homologous sequences in a direct orientation, resulting in the excision of the intervening sequence. (3) Intramolecular CO between two homologous sequences in an inverted orientation, resulting in the inversion of the internal fragment. (4) and (5) Inter-chromosomal CO, depending upon the orientation of the homologous sequences with respect to their centromeres (blue or red circles); this process generates a translocation (4) or a dicentric and an acentric chromosome (5).

HR is initiated through the invasion of a duplex DNA by a homologous single-stranded molecule, which then primes DNA synthesis (Figure 1B). The strand invasion, promoted by RecA/Rad51, is able to occur with homologous sequences containing few heterologies (although the divergences should be limited), thus generating heteroduplex DNA molecules bearing mismatches (Figure 1B). The repair of these mismatched structures can transfer sequence polymorphisms and modify the genetic information of the recipient molecule, resulting in an apparent mutagenic event. Additionally, the DNA synthesis initiated by the invading strand (Figure 3A) can duplicate a sequence that was absent in the donor molecule and thereby transfer this genetic information, resulting in modifications of the original recipient DNA sequence. Moreover, the resolution of the HR intermediate (Holliday junctions) can facilitate the exchange of adjacent sequences, leading to genetic rearrangements. Thus, both GC and CO intrinsically possess the capacity to modify genetic information. This has been used to target gene replacement and gene correction using exogenous DNA. Note that when involving identical sequences (for instance sister chromatids exchange: SCE), HR does not impact the genetic information. However, unequal SCE can lead to sequence duplication or deletion (Figure 3B). One can object that unequal SCEs should be less frequent than equal SCEs (Gonzalez-Barrera et al., 2003). Therefore, genome stability should not be strongly impacted by SCEs. In contrast, when involving repeated sequences (which are not identical) dispersed throughout the genome (non-allelic recombination, NAHR), HR can affect the genetic information (see below). Note that, if the final product of an equal SCE is error-free, this is not due to the accuracy of the HR process, but to the fact that the DNA are identical (indeed HR can efficiently processes with imperfectly homologous sequences) and because associated mechanisms orientate such kinds of events: 1-HR is restricted to the S and G2 phases (which correspond to the cell cycle phases presenting sister chromatids) and 2-the tight cohesion of the sister chromatids, through the cohesins complex, orientates the event to an equal SCE. Thus, the structure of the DNA and accessory associated mechanisms, rather than HR itself, favor such an error-free event. In addition, HR can initiate mutagenic DNA synthesis even when the interacting DNA molecules are fully identical such as sister chromatids (see discussion below). Finally, we can point out that, in yeast as well as in mammalian cells, spontaneous SCE have been described to be largely independent of the main actors of HR (Rad51, Rad52, Rad54), in contrast with induced SCE (Dronkert et al., 2000; Fasullo et al., 2001; Lambert and Lopez, 2001; Dong and Fasullo, 2003). Noteworthy, at meiosis, which aims at creating genetic diversity, equal SCEs are repressed and HR between homologous chromosomes (which are not identical) is favored. Therefore, in this situation, HR is used to generate genetic diversity.

Thus, in the cases discussed above, associated processes, rather than the HR machinery itself, in fact control the accuracy of the final outcome of HR.

### Genetic alterations through GC and/or CO

Gene conversion is able to transfer genetic information in a non-reciprocal manner between two hetero-alleles, resulting in loss of heterozygosity; gene conversion can also transfer one stop codon from a pseudogene to a related coding sequence, leading to its extinction (Figure 3C) (Amor et al., 1988; Fusco et al., 2012). Moreover, crossing over between repeated sequences that are dispersed throughout the genome (non-allelic HR) could lead to genomic rearrangements, such as translocations, deletions, amplifications and inversions (Figure 3D). These models account for genome rearrangements responsible for different human pathologies, attesting to the existence of these processes *in vivo* (Purandare and Patel, 1997; Chen et al., 2007; Fusco et al., 2012).

### HR-mediated genome rearrangements by BIR and non-allelic HR

In *Saccharomyces cerevisiae*, using an intron-based chromosomal translocation assay, it has been reported that DSB-induced translocation occurs via triparental recombination events. A short homologous sequence in the third chromosome serves as a bridge template for recombination events occurring between two non-homologous chromosomes. These events give rise mainly to reciprocal translocations that require the HR proteins Rad52 and Rad51 and the BIR-specific protein Pol32. Rad59 and Srs2 are also required, although to a lesser extent, whereas KU70 plays no role. These data suggest that BIR-mediated triparental recombination could be a major mechanism for chromosomal translocations in eukaryotic cells (Schmidt et al., 2006; Ruiz et al., 2009). Using a newly designed substrate for the analysis of DSB-induced chromosomal translocation, the group of Aguilera shows that Mus81 and Yen1 endonucleases promote BIR, thus causing non-reciprocal translocations. These endonucleases, as well as Slx4, promote replication template switching during BIR, thus participate in the generation of complex rearrangements when repeated sequences dispersed throughout the genome are involved (Pardo and Aguilera, 2012).

BIR can also induce genome instability in mammalian cells. It was recently reported that replicative stress induced by the overexpression of cyclin E in human cells led to copy number alteration (CNA). One third of these genome alterations (duplications less than 200 kb) have been attributed to BIR events or to microhomology-induced replication (MMBIR), a BIR-related mechanism (see below). The depletion of Pol D3, which encodes a subunit of pol delta, decreases the frequency of these events. The authors propose that BIR repair of damaged replication forks might explain the presence of segmental genomic duplication in human cancers. The larger amplification (>200 kb) and deletion observed after the overexpression of cyclin E may arise from other repair mechanisms, such as non-allelic HR (Costantino et al., 2014).

Replication fork arrest has also been reported to promote non-allelic HR between repeated sequences. In budding yeast, a reduced level of replicative polymerases, which can potentially alter the progression of replication forks, leads to recombination between an inverted Ty element and translocation (Lemoine et al., 2005, 2008). A more direct connection between fork arrest and HR-mediated genome rearrangements has been established in fission yeast, in which the block of a single replication fork leads to translocation and genomic deletion that results from HR between repeated sequences (Lambert et al., 2005; Iraqui et al., 2012). Such chromosomal rearrangements are a direct consequence of replication restart at unbroken forks by HR and not a consequence of failure in restarting forks and subsequent aberrant processing (Mizuno et al., 2009).

Given the potential role of HR in mediating chromosomal rearrangement, factors that prevent non-allelic HR might thus be considered as factors protecting against homology-mediated genomic instability. For example, increasing the distance between repeated sequences reduced the frequency of non-allelic HR (Lichten and Haber, 1989; Godwin et al., 1994). In fission yeast, CENP-B factors facilitate fork passage across LTR repeats that are prone to fork blockage. In the absence of CENP-B, LTR behaves as an HR hot spot prone to deletion events (Zaratiegui et al., 2011).

### HR-induced mutagenesis

Mutagenesis associated with HR was first reported in *E. coli* (Cairns and Foster, 1991; Harris et al., 1994; Rosenberg et al., 1994). Repair of DSBs by HR in *E. coli* is non-mutagenic in unstressed cells, but under stress, switches to a mutagenic mode that is activated by stress responses (Ponder et al., 2005; Shee et al., 2011). This mutagenic repair of DNA breaks requires proteins that mend DSBs by HR, error-prone DNA polymerases, activation of SOS DDR, the controlled general and starvation stress response (RpoS), and a membrane protein stress response (RpoE), that promotes spontaneous DNA breakage in some DNA regions (Gibson et al., 2010). RpoS controls the switch that changes the normally high-fidelity process of DSBR *via* HR to an error-prone one. In this pathway, three steps are required: (1) DSB repair initiated by HR proteins (RecBCD, RecA); (2) the activation of SOS upregulates PolIV/DinB error-prone DNA polymerase; and (3) a second stress that activates RpoS, which allows Pol I, II, V, and/or PolI to participate in break repair instead of (or in addition to) the high fidelity DNA polIII (for review Rosenberg et al., 2012). This mechanism limits genetic instability to the stress response and to regions near a DSB, and therefore produces localized mutations rather than dispersed mutations. This could be an important evolutionary strategy, both for the minimization of deleterious mutations in cells that acquire a rare adaptive mutation and also for concerted evolution within genes and gene clusters (reviewed in Rosenberg et al., 2012).

Using HO-generated DSBs, it has been shown that mitotic recombination is mutagenic, which has been referred to as break-repair-induced mutation (BRIMs) (Strathern et al., 1995; Rattray et al., 2002; and reviewed in Abdulovic et al., 2006). Both error-prone DNA synthesis associated with DSB repair and stretches of ssDNA might account for BRIMs. During DSB repair, the DNA-end-resection machinery generates intermediates containing ssDNA that are highly sensitive to mutations due to the activity of the trans-lesion synthesis DNA polymerase Zeta (Yang et al., 2008). In addition, it has recently been shown that the DNA synthesis step during elongation of the invading strand is highly mutagenic in *Saccharomyces cerevisiae*, with the mutation rate increasing by up to 1400-fold, and exhibits a mutation signature (primarily microhomology-mediated inter-strand template switching). These mutations result from errors that are made by Polδ and Polε (Hicks et al., 2010). Importantly, HR can be mutagenic even when involving a long tract of DNA synthesis. Indeed, BIR, one of the HR-type processes that are thought to restart replication forks, duplicates DNA over a long distance, even to the end of the chromosome arm, by establishing a replication fork-like structure (Figure 1B). Strikingly, in *Saccharomyces cerevisiae*, DNA synthesis that is induced through BIR is highly inaccurate over the entire path of the replication fork. The high level of mutation results from the combinatorial effects of an increase of the nucleotide pool induced by the DDR, the uncoupling of DNA synthesis with mismatch repair, and the exposure of ssDNA (Deem et al., 2011). Recently, BIR has been proposed to proceed via a migrating D-loop mediated by the helicase Pif1. The migration of the D-loop results in the extrusion of the synthesized DNA and the exposure of a long stretch of ssDNA, which can become a hot spot for lesions leading to mutations (Saini et al., 2013; Wilson et al., 2013). In support of this hypothesis, BIR-induced mutations are largely dependent on Pif1 (Saini et al., 2013; Wilson et al., 2013).

One essential role of HR is to reactivate arrested replication forks. In *Schizosaccharomyces pombe*, this process is error-prone. As mentioned above, replication restart by HR mediates non-allelic HR. More surprisingly, it also leads to small deletions and duplications flanked by micro-homology. Indeed, replication forks restarted by HR are associated with error-prone DNA synthesis, liable to template switch events at micro-homologies (Iraqui et al., 2012). When progressing across small inverted repeats or palindromes, forks recovered by HR are prone to generate large chromosomal inversions (Mizuno et al., 2013).

### Anti-HR mechanisms for protection against genetic instability and cell toxicity

One mechanism avoiding potential genetic instability promoted by HR is to orientate it to equal SCEs, while unequal SCEs are mutagenic (see Figure 3B). Indeed, sister chromatids are identical, thus GC cannot transfer mutation and CO will not have any genetic impact. This is done by associating two processes (as discussed above): (1) restriction of HR in S and G2 phase and (2) the cohesion of the sister chromatids.

Excess HR can also lead to the accumulation of HR intermediates, which generates genomic instability and cell death (Gangloff et al., 2000). Thus, HR is a double-edged sword; on the one hand, it protects against genetic instability, but on the other hand, it can trigger cell lethality as well as profound genomic rearrangements and point mutations. Therefore, the HR process should be tightly controlled to avoid unnecessary HR events. Helicases, by destabilizing abortive HR intermediates, protect against the genomic instability generated by HR (reviewed in Barber et al., 2008; Chu and Hickson, 2009; Bernstein et al., 2010). Additionally, it has been proposed that restricting the initiation of unscheduled HR can also prevent against the accumulation of such toxic HR intermediates. In mammalian cells, this protective role against excessive HR initiation has been proposed for p53, Bcl-2, and AKT1 (Bertrand et al., 2004; Plo et al., 2008; Guirouilh-Barbat et al., 2010; Laulier et al., 2011a; Laulier and Lopez, 2012).

Of note, the fact that protective systems have evolved to counteract excess HR underlines the potential risks of this pathway.

### Relationships with chromothripsis and kataegis

The classical theory of cancer development proposed that cells gradually and randomly accumulate mutations and rearrangements that increase their survival (reviewed in Stratton et al., 2009). However, recent studies have revealed that critical aspects of cancer development can occur on a much shorter timescale. In a process called chromothripsis (from the Greek chromos for chromosome and thripsis, shattering into pieces), tens to thousands of genomic rearrangements occur in one cellular crisis (Berger et al., 2011; Stephens et al., 2011). In kataegis, mutations accumulate in hotspots of hundreds of bases to megabases in a single cell cycle (Nik-Zainal et al., 2012; Roberts et al., 2012). Interestingly, both processes are linked to DSB repair events.

In chromothripsis, cells undergo tens to thousands of genomic rearrangements clustered into discrete subchromosomal territories, as first described in a small set of tumors (Berger et al., 2011; Stephens et al., 2011) and subsequently observed in a wide variety of tumors (Kloosterman and Kuipers, 2011; Magrangeas et al., 2011; Lapuk et al., 2012; Molenaar et al., 2012; Rausch et al., 2012). What causes such a dramatic remodeling of the genome is still unknown. However, the implicated regions are sharply circumscribed and this suggests that the original DNA damage occurs during mitosis when DNA is highly condensed. Although several mechanisms have been suggested to explain the clustered rearrangements, the most plausible cause is replicative stress on regions difficult to replicate (e.g., fragile sites). In particular, replication intermediates that do not expose long stretches of ssDNA and therefore do not activate the checkpoints allow cells to enter mitosis in their presence (Chan et al., 2009). A recent study suggested that chromosome shattering might arise from an error in mitotic chromosome segregation that leads to the production of micronuclei (Crasta et al., 2012). These micronuclei are at high risk for the integrity of the genome. First, they exhibit a defective DDR and delayed or defective DNA repair (Terradas et al., 2009, 2012; Crasta et al., 2012). Second, most micronuclei replicate more slowly than the major nucleus and therefore most micronuclei are still replicating when the major nucleus is already in the G2 phase (Crasta et al., 2012). Finally, entry in mitosis when the micronucleus is still replicating is associated with a massive induction of DSBs (Crasta et al., 2012).

**Figure 4:**
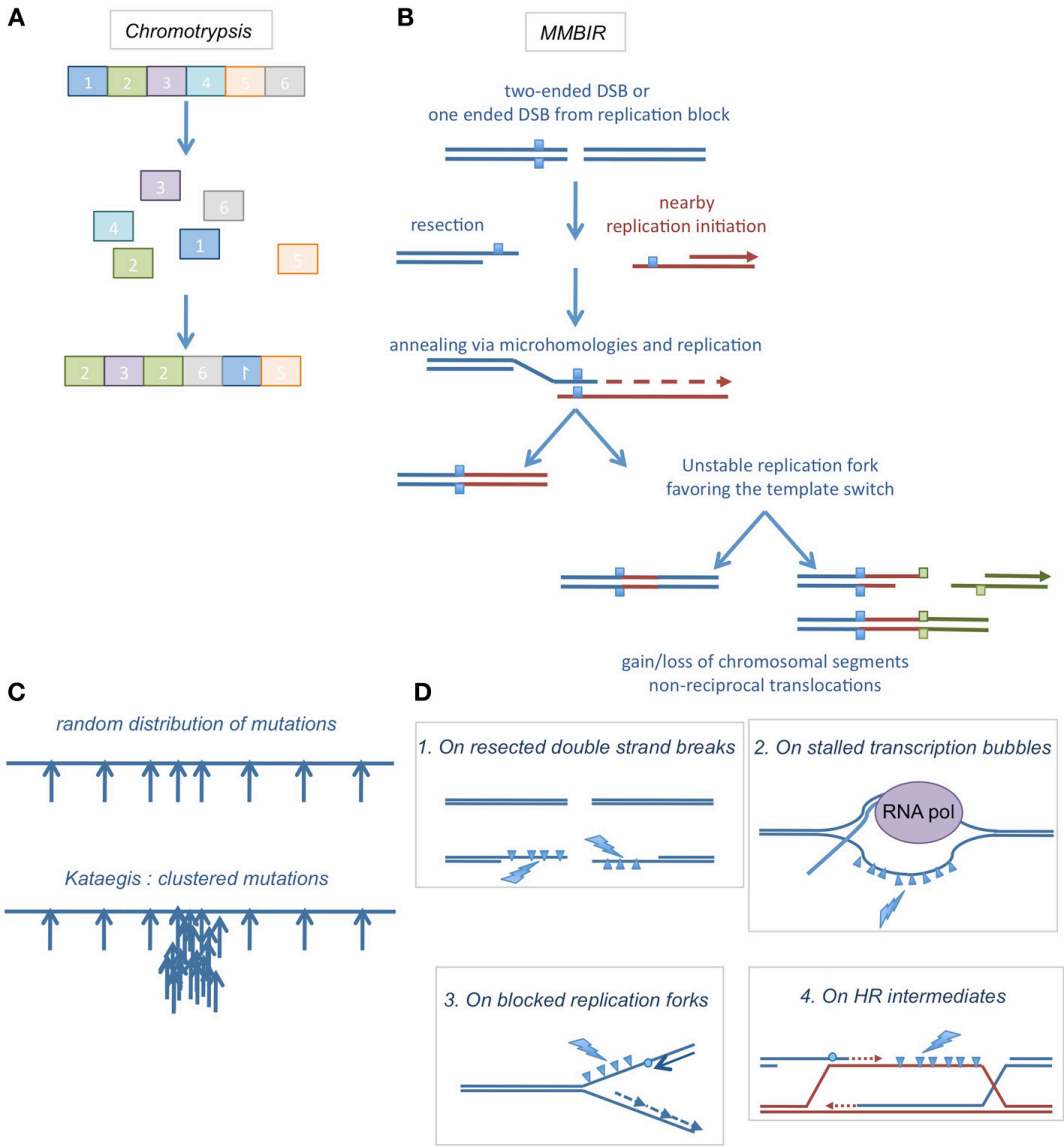
**(A)** Chromothripsis. Chromosomal shattering into pieces and abnormal re-ligation events, resulting in intra- or inter-chromosomal rearrangements. **(B)** A suggested model for chromothripsis occurrence, the MMBIR (microhomology mediated break induced replication). A DNA double strand end is resected to generate a 3′ overhang that will anneal with microhomologies elsewhere in the genome to initiate replication. This mechanism can lead to more complex rearrangements if it is coupled to multiple cycles of template switches. **(C)** Kataegis. When mutations are expected to be distributed randomly in the genome (upper cartoon), clustered mutations were found in the genomes of several cancers (lower cartoon). **(D)** Where kataegis occurs. These clustered mutations were at least in part correlated with the action of DNA deaminases of the APOBEC family, which deaminate cytosines on ssDNA areas found on resected DNA ends (1), stalled transcription bubbles (2), blocked replication forks (3), or HR intermediates (4).

The DNA repair machinery then reassembles the chromosomal pieces in a disordered fashion (see example in Figure 4A). The possible mechanisms of chromosome reassembly first implicated NHEJ and A-EJ because the junction sequences exhibited tracts of microhomology, as well as insertions or deletions of variable sizes (Rausch et al., 2012; Stephens et al., 2012). However, these mechanisms can account for the loss of genetic information but not for amplification of some genomic regions (Magrangeas et al., 2011; Rausch et al., 2012; Stephens et al., 2012). Replication-based repair pathways are more plausible, accounting for both genomic gains and losses. A hybrid of replication-independent mechanisms and replication-dependent processes has been proposed to explain the complex rearrangements found in chromothripsis, the MMBIR (microhomology-mediated break induced replication) (Figure 4B) (Hastings et al., 2009; Liu et al., 2011) associated with a specific mechanism linked to replication block, FoSTeS (for Fork Stalling and Template Switching) (Lee et al., 2007; Zhang et al., 2009). These processes begin with the conversion of a DSB (or a replication fork stall) in a ssDNA 3′ stretch. This free 3′DNA end can then anneal using a region of micro-homology on a ssDNA region exposed on an adjacent replication fork. Replication can then occur. However, such replication forks are weakly processive and can undergo several rounds of template switching, generating complex rearrangements with deletions, amplifications and non-reciprocal translocations. The use of this low fidelity repair process to manage the high level of DSBs generated during chromothripsis could be explained by the overwhelming of reliable repair processes and DDRs. It is worth mentioning that not all chromothripsis events are explainable by FoSTeS or MMBIR; some of them might be the result of chromosome shattering followed by NHEJ or A-EJ.

In 2012 has been reported the occurrence of somatic localized mutation hotspots in tumor genome, called kataegis (from the Greek for thunderstorm) (Nik-Zainal et al., 2012; Roberts et al., 2012). This mechanism was then observed in a broad range of cancers (Alexandrov et al., 2013). In kataegis, mutations accumulate rapidly at somatic mutation hotspots (Figure 4C) at a critical step of tumorigenesis. Several mutation signatures were identified, particularly mutations on guanines and cytosines.

The mutation pattern matched the signatures of the RNA- and DNA-editing deaminases of the AID/APOBEC family that act on ssDNA molecules. Indeed these enzymes deaminate cytosines and generate uracils that are a substrate for Base Excision repair, generating abasic sites, causing C-to T-transitions or driving polymerase eta misincorporations. Before kataegis was described, genome sequencing studies had revealed that many cancers have somatic mutations dominated by C-to-T transitions (Sjoblom et al., 2006; Greenman et al., 2007; Jones et al., 2010; Berger et al., 2011; Kumar et al., 2011; Parsons et al., 2011; Stransky et al., 2011; Taylor et al., 2013) and that overexpression of APOBEC1 was associated with cancer development (Yamanaka et al., 1995) when overexpression of APOBEC3A induced genomic damage and mutations (Stenglein et al., 2010; Landry et al., 2011; Suspene et al., 2011). The implication of APOBEC deaminases in kataegis was validated by several groups in yeast models (Taylor et al., 2004; Chan et al., 2012; Roberts et al., 2012) and in human cells (Burns et al., 2013), where overexpression of APOBEC3B was correlated with an elevated level of mutations in breast tumors and cell lines. Knockdown experiments showed that endogenous APOBEC3B was responsible for increased mutation frequencies and C-to-T transitions when APOBEC3B overexpression induced DNA damage and C-to-T mutations in human cells.

As mentioned above, AID/APOBEC enzymes deaminate only cytosines in ssDNA. It was therefore proposed that these deamination reactions could occur on stabilized ssDNA stretches formed on stabilized transcription bubbles or after the occurrence of DSBs or replication fork blockage (Figure 4D). In the last case, the uncoupling between helicases and polymerases generates and stabilizes long patches of ssDNA.

Interestingly these strand coordinated clusters of mutated cytosines or guanines were often localized next to chromosome rearrangement breakpoints and extended up to 200 kb (Roberts et al., 2012) suggesting that they were correlated to the occurrence of DSB and DSB repair pathways generating ssDNA stretches, like HR (see Figure 1). The correlation between DSB induction and kataegis was confirmed in yeast treated with alkylating agents (Roberts et al., 2012) or even more directly, in yeast where DSB were induced by the meganuclease I-SceI (Taylor et al., 2013): In these studies, the authors observed a strand bias in the mutations observed. Cytosines were preferentially mutated on the 5′ side of a DSB and guanines on the 3′ side of the DSB. As resection only occurs in the 5′ to 3′ direction, this pattern in mirror was correlated to the generation of ssDNA stretches in Homology directed repair. It is noteworthy that HR is not the only mechanism leading to ssDNA stretches that are a template for kataegis; uncoupled replication forks that expose long stretches of ssDNA are also a template for deaminases (Roberts et al., 2012).

The association of the timescale between kataegis and chromothripsis suggests that both could occur simultaneously at certain chromosomal regions, resulting in an even more catastrophic event for the cell.

## The importance of being versatile

HR is versatile because it tolerates limited divergences between the interacting partners. Remarkably, this capacity to modify genetic information has been used by cells to generate beneficial genetic diversity. HR has therefore been implicated in numerous essential biological processes, from molecular evolution to DNA repair and meiotic differentiation, and is also relevant to targeted gene replacement.

At meiosis, HR ensures that allele mixing creates genetic diversity. In chickens, gene conversion of the expression allele with pseudo-genes generates the complexity of the immune repertoire (Reynaud et al., 1987).

In pathogens, antigenic variation is a widely used strategy for immune evasion. Gene conversion is a prominent system for antigenic variation through recombination between one silent copy of a gene and the expressed copy, resulting in the formation of a chimeric gene. Several pathogens, such as *Trypanossoma brucei, Anaplasma marginale, Borrelia burgdorferi, Helicobacter pylori*, and *Neisseria gonorrhoeae*, use this strategy (Palmer and Brayton, 2007; Stockdale et al., 2008; Wisniewski-Dye and Vial, 2008). For example, trypanosomes are coated with a variant surface glycoprotein (VSG). Antigenic variation involves switches in the composition of the VSG coat driven by gene conversion between the expressed allele and an archive of silent VSG genes (Marcello et al., 2007; Morrison et al., 2009). In *Candida albicans*, recombination generates homozygous hyperactive alleles conferring resistance to antifungals (Coste et al., 2006).

HR is a driving-force in the evolution of multi-gene families; crossovers leading to unequal exchanges between sister chromatids are responsible for variation in the repetition of duplicated sequences. During evolution, most duplicated sequences diverge; the genes of one species derived from a common ancestor are paralogs. Due to selective pressure, there are generally fewer divergences between homologous genes of two different species (orthologs) than between their respective paralogs. However, in some families of repeated genes, the divergence between the duplicated units is less significant within one species than when compared to a different species, even one that is evolutionarily close. In this case, the duplicated genes did not evolve independently but instead co-evolved; this phenomenon is called “concerted evolution” (reviewed Arnheim, 1983; Liao, 1999). Gene conversion is the driving force behind homogenization of duplicated sequences, and therefore of concerted evolution. Concerted evolution is a universal biological phenomenon that occurs in bacteria, yeast, plants and animals. Because HR should be tightly controlled, some processes exist to limit it. Indeed, sequence heterologies block gene conversion and should therefore be barriers to concerted evolution; it has been suggested that introns, which can interrupt the length of sequence homology without affecting the function of the encoded protein, can be protective barriers against HR between repeated sequences, thereby favoring the maintenance of the structural organization of the genome (Kourilsky, 1983; Kricker et al., 1992). In this context, it is tempting to speculate that introns are an evolutionary force antagonistic to concerted evolution, directing evolution toward the divergence of repeated sequences.

## Up- and down-regulation of HR in cancer

Genetic instability is a hallmark of cancer cells. Both inhibition and stimulation of HR have been reported in tumors or cancer-prone situations. This is consistent with the duality of HR, and this underlines that inhibition as well as stimulation of HR confer increased risks of genetic instability. More precisely, both down- and up-regulation of the recombinase RAD51 affects genomic stability.

For instance, the expression of a non-lethal dominant negative form of RAD51 in cells injected into nude mice favors tumor take and growth (Bertrand et al., 2003). The overexpression of RAD51 stimulates HR (Vispé et al., 1998; Huang et al., 1999; Lambert and Lopez, 2000) and induces a strong chromosome instability (Richardson et al., 2004), underlying the potential risks of excess HR. These data highlight the importance of tight control of the level of HR.

### HR defects associated with predisposition to cancers

Most of the mutations responsible for familial breast or ovarian cancers affect genes that control HR and/or the replication/HR interface directly or indirectly (Walsh and King, 2007; Walsh et al., 2011). The two genes most often mutated, BRCA1 and BRCA2, are two major players in HR (Moynahan et al., 1999, 2001). This overrepresentation of genes involved in the response to DNA damage and the communication between replication and recombination suggests the importance of these specific metabolic pathways in the etiology of breast cancer and raises the question of characteristics common to the causation of sporadic and hereditary breast cancer. Several studies have reported the hyperactivation of the oncogenic kinase AKT1 in 40-60% of sporadic breast cancers and in 40% of sporadic ovarian cancers (Sun et al., 2001; Yang et al., 2006; Plo et al., 2008). It must be noted that PTEN, one of the genes mutated in familial breast cancer, is an antagonist of AKT1. Several studies have shown connections between AKT1 and responses to DNA damage (for a review, see Guirouilh-Barbat et al., 2010). In particular, overexpression of AKT1 induces the sequestration of BRCA1 and RAD51 in the cytoplasm, leading to the inhibition of HR (Plo et al., 2008; Plo and Lopez, 2009). Taken together, these data underline the importance of HR in protection against breast cancer and reveal the AKT1 signaling pathway as a missing link between hereditary and sporadic breast cancers.

Other examples of HR inhibition exist in situations of predisposition to cancer. For example, Bc1-2 is an inhibitor of the intrinsic pathway of apoptosis induction, and its activation confers a predisposition for lymphomas. Bc1-2 was initially found to be overexpressed in B cell lymphoma with the recurrent translocation *t*_(14:18)_, but it is also overexpressed in numerous tumors. Remarkably, overexpression of Bc1-2 leads to the relocalization of BRCA1 in endomembranes (endoplasmic reticulum, mitochondria), resulting in an inhibition of HR (Laulier et al., 2011a; and reviewed in Laulier and Lopez, 2012).

### Stimulation of HR in cancer

Conversely, there are also situations associating a predisposition for tumors and hyper-recombinogenic phenotypes.

For example, in Bloom syndrome, there is a greatly elevated predisposition to spontaneous tumors in all tissues. Bloom syndrome results from the inactivation of the BLM protein, a member of the RecQ helicase family, that plays an important role in the resolution of HR intermediates, in the processing of blocked replication forks, and at the initiation of DNA double strand break repair (Bernstein et al., 2010; Grabarz et al., 2013). Cells from patients afflicted with Bloom syndrome show increased levels of exchange between sister chromatids and hyper-recombination phenotypes (reviewed in Chu and Hickson, 2009).

The tumor-suppressing p53 gene is the most frequently mutated gene in all types of cancers. It has been shown that the p53 protein represses HR; cells deficient in p53 show a hyper-recombination phenotype (for a review, see Bertrand et al., 2004).

The fusion oncogene BCR/ABL derives from the translocation of the cABL gene from chromosome 9 to the BCR gene locus on chromosome 22: Philadelphia chromosome *t*_(9:22)_. This translocation is present in chronic myelogenous leukemia (CML) patients and in many acute lymphocytic leukemia patients. The BCR/ABL fusion proteins (p230, p210, or p185) exhibit constitutive tyrosine kinase activity. The resistance of BCR/ABL tumors to DNA damage induced by therapeutic drugs depends on the kinase activity of the fusion protein. The expression of BCR/ABL increases the intracellular level of RAD51 protein by different mechanisms (Slupianek et al., 2001). First, signaling from the BCR/ABL src homogy-3 (SH3) and SH2 domains stimulates RAD51 transcription *via* the activation of the signal transducer and activation transcription 5 (STAT5). The transcription of the paralogs RAD51B, RAD51D, and XRCC2 is also stimulated, whereas transcription of RAD51C and XRCC3 is decreased. Second, BCR/ABL inhibits caspase-3 activation and thus RAD51 protein degradation. Indeed, BCR/ABL stimulates HR between tandem repeat sequences. Additionally, BCR/ABL interacts with RAD51 and results in a high level of constitutive Tyr315 phosphorylation. This Tyr315 phosphorylation and RAD51-dependent HR seem to control resistance to cisplatin and mitomycin C (Slupianek et al., 2001). BCR/ABL expression inhibits DNA-PK activity, which is involved in non-homologous end joining, a competitor pathway to HR for DNA DSB repair (Deutsch et al., 2001). This suggests that the regulation of the balance between HR and NHEJ can be modified by BCR/ABL.

## Conclusions

### HR: A double-edged sword

Regulation of HR should permit the maintenance of genomic stability, allowing genetic diversity but avoiding genetic instability. Depending on the structure of the interacting DNA partners, GC and CO intrinsically possess the capacity to generate genetic variability/instability. In addition to cell cycle regulation, which inhibits HR in the G1 phase and restricts it at the S-G2 phase (during which the sister chromatids are generated) and the tight cohesion of the sister chromatids that orientates exchange to equal SCE, several additional mechanisms repress HR: mismatch repair, helicases, and p53. Defects in these systems are associated with genome instability and cancer predisposition. The fact that living organisms develop strategies to repress HR underlines the potential dangers of HR excess. Indeed, excess HR does generate mutagenesis and genomic rearrangements. These capacities have been used by cell to generate beneficial genetic diversity, but conversely, many pathological rearrangements are explained by accidental HR.

Strikingly, ablation of replication origins in *Archaea* bacteria results in faster growth thanks to the initiation of replication by HR (Hawkins et al., 2013). This raises the question as to why organisms use replication origins to duplicate the entire genetic material, instead of HR. Considering the potential risks of HR both for the accuracy of DNA replication and for genomic architecture, the choice of replication origins should ensure a more stable and accurate duplication through generations; note that this should allow for the maintenance of the minimum common genomic structure defining a given species. In contrast, HR, especially CO, would lead to highly rearranged DNA in offspring, resulting in genetic separation between ancestors and progeny. While genome modification is a driving force for evolution giving opportunity to generate individual genetic diversity, an intergenerational maintenance of the genome should facilitate speciation.

### Accuracy of HR vs. NHEJ: the world turns upside down

In many scientific reports (publications, reviews, thesis dissertations, conferences), HR is claimed to be error-free, whereas NHEJ is said to be error-prone. However, the two processes share similarities:

- Both HR and NHEJ are required for genome stability maintenance.
- Both are involved in processes generating genome diversity.
- Both can generate genome rearrangements.
- In both cases, the structure of the DNA molecules determines the final product.

However, they also show differences:

- In contrast with the common view, HR contains the intrinsic capacity to modify genetic material through GC and CO (this has been used to generate genetic diversity in meiosis or V(D)J recombination in chicken) and by promoting error-prone DNA synthesis, while NHEJ is not intrinsically error-prone and can join fully complementary DNA ends mainly in a faithful manner (for review see Betermier et al., 2014).

Therefore, HR, which can generate genetic alteration, should be tightly control to limits its potential danger and to lead to accurate outcomes. However processes aiming at generating genetic diversity take advantage of these intrinsic capacities of HR.

### Conflict of interest statement

The authors declare that the research was conducted in the absence of any commercial or financial relationships that could be construed as a potential conflict of interest.
